# Cell migration into the damaged brain mediated by increased cell adhesion

**DOI:** 10.1038/s44321-024-00075-5

**Published:** 2024-05-24

**Authors:** Jemima Becker, Francis Szele

**Affiliations:** Department of Physiology, Anatomy and Genetics, University of Oxford, Oxford, OX1 3PT UK

**Keywords:** Neuroscience, Pharmacology & Drug Discovery

## Abstract

F. Szele and J. Becker discuss a new mechanism of neuronal migration in healthy and injured brain and a promising therapeutic potential of a neuraminidase inhibitor for the treatment of brain injury as reported by K. Sawamoto and colleagues, in this issue of *EMBO Mol Med*.

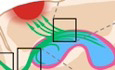

## Teaser

A new paper published in *EMBO Molecular Medicine*, Matsumoto et al (2024), elegantly shows that polysialic acid (PSA), has a new function—inhibition of migration. Molecular control over cell migration during brain development is essential for proper brain growth and positioning of neurons. Newborn cells frequently migrate long distances and in circuitous routes to their final destinations. Although many molecular regulators have been found that direct cell migration, the fundamental questions of how and why the cells exhibit such long-distance journeys is not well understood. This is especially true in the context of the rostral migratory stream (RMS) in the postnatal brain.

**Figure 1:**
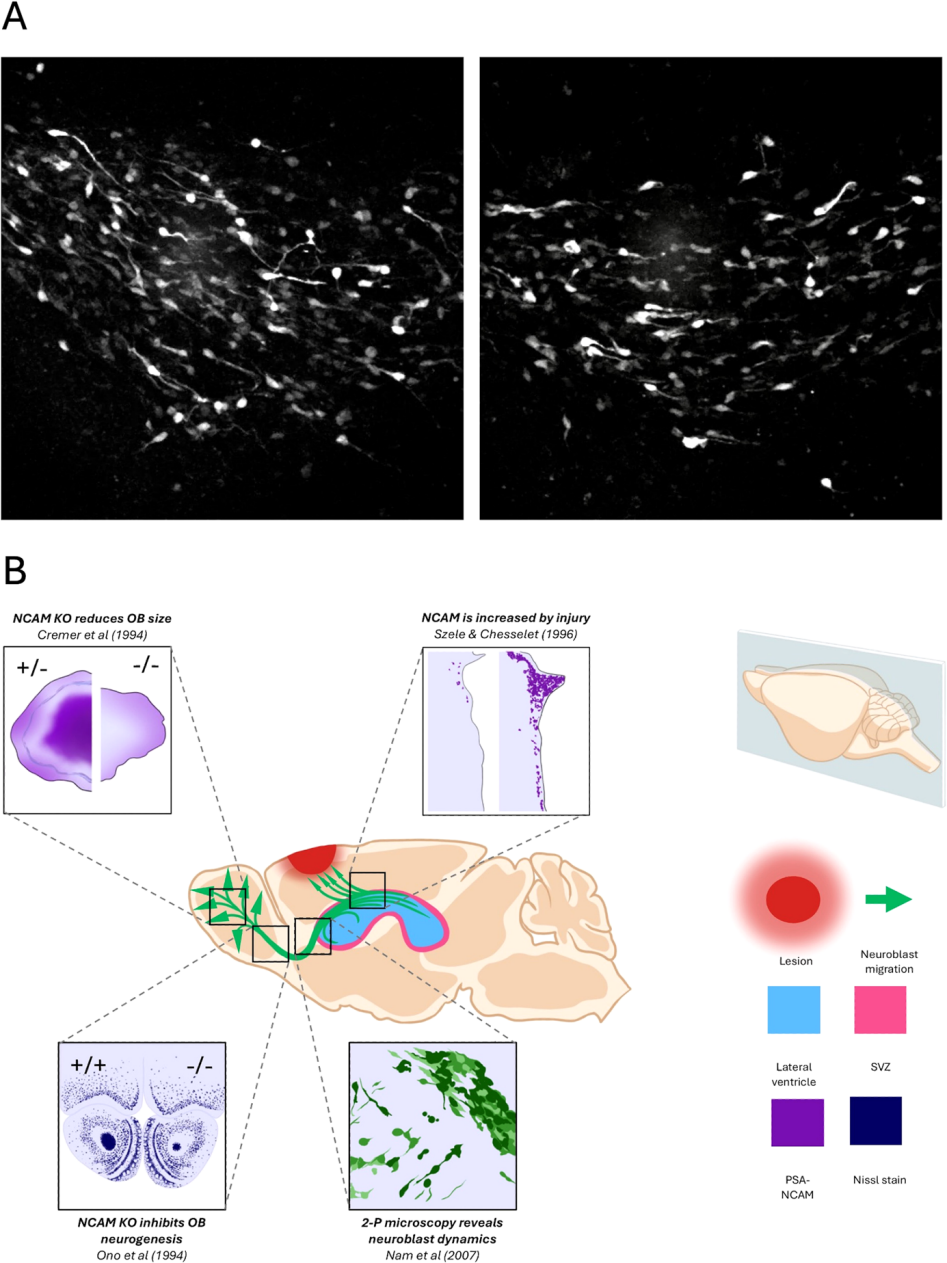
Neuroblast migrations. (**A**) Nestin-GFP+ cells migrating in the rostral migratory stream, viewed by 2-Photon microscopy. Note that this mouse did not label all RMS neuroblasts allowing easy visualisation of individual cells. Chain migration is better seen with the Dcx-GFP mice in which all neuroblasts were labelled. Adapted with permission from Nam et al (2007). Video available on YouTube at: https://www.youtube.com/shorts/i_N6NS5etDI. (**B**) Summary of selected evidence regarding SVZ migration dynamics and PSA-NCAM. The mouse brain is shown in sagittal view, with the SVZ illustrated in pink, the lateral ventricles in blue, and the migration of SVZ-derived neuroblasts in green.

The RMS is composed of newborn neuroblasts migrating from their birthplace in the subventricular zone (SVZ) lining the lateral ventricles and moving to the olfactory bulbs (Fig. 1A) (https://www.youtube.com/shorts/i_N6NS5etDI). This fundamental aspect of the SVZ stem cell niche, migration, has been known since Joseph Altman’s seminal work on the RMS in the 1960’s, yet over six decades later it is still one of the least studied aspects of neurogenesis. This lack of understanding is weird since neuronal migration is as important as the more popular aspects of brain development such as stem cell quiescence versus activation. The development of high-resolution live-imaging techniques such as 2-photon microscopy have allowed further insight into fundamentally important details of neuroblast dynamics (Fig. 1B) (James et al, 2011; Nam et al, 2007) and recent 3-photon studies have shown that the SVZ/RMS can be imaged in live animals (Sun et al, 2022).

A key aspect of the RMS with clinical relevance is that both human and animal neuroblasts migrate from the SVZ to brain injuries and disease induced lesions (Fig. 1B) (Chang et al, 2016). Neuroblasts and their glial counterparts have been shown in many cases to migrate towards traumatic brain injury, stroke, multiple sclerosis lesions and other pathological regions. Important animal work has shown that reducing or removing this capacity negatively affects the brain’s ability to repair itself (Jin et al, 2010).

Despite this clinical potential, it has been difficult to acquire consistent evidence for RMS migration to the olfactory bulbs after the first year of life in humans. However, accumulating evidence suggests that in humans SVZ cells migrate to a variety of brain regions even in health. Frisen’s cold war radioactive carbon dating suggests that in humans, SVZ-derived neuroblasts migrate not into the OB but into the caudate putamen (Ernst et al, 2014). Work from the Alvarez-Buylla lab has also indicated novel human-specific migration routes from the SVZ including the ventromedial pre-frontal cortex and the entorhinal cortex (Nascimento et al, 2024; Paredes et al, 2016). These cellular migrants may be vulnerable to gene mutations or environmental toxins and become deleterious to normal function. These novel migration routes suggest that even more research is needed on the RMS.

There are many questions remaining to be addressed concerning SVZ cell migration (James et al, 2011) including the following.

1. How similar or novel are mechanisms of neuroblast migration across brain regions and different species?
2. What chemoattractants versus chemorepellants regulate the routes taken?
3. What determines cell speed?
4. What are the “motogens” that get cells going?
5. What are the molecules that tell them when and where to stop?
6. Does emigration from the SVZ to ectopic injury locations require molecular mechanisms not normally at play or does it simply co-opt extant mechanisms in novel configurations?
7. Could SVZ neuroblasts be genetically engineered to migrate to lesions and acquire region-appropriate fate choices?

Neural cell adhesion molecule (NCAM) was the first molecule to show important functions in RMS neuroblast migration (Fig. 1B). Thirty years ago Cremer and colleagues demonstrated that NCAM deletion significantly reduces RMS migration (Cremer et al, 1994). NCAM is polysialylated (PSA-NCAM) and the PSA residues are thought to decrease adhesion in RMS neuroblasts, thereby facilitating migration (Fig. 1B) (Ono et al, 1994). PSA-NCAM labels all RMS neuroblasts, and only neuroblasts, and thus is an excellent molecular target and readout of neurogenesis. The first study examining SVZ PSA-NCAM expression in pathology detected an enormous increase in expression after cortical injuries (Fig. 1B) (Szele and Chesselet, 1996).

A new manuscript published in this edition of EMBO Molecular Medicine, explores the functional role of PSA-NCAM in repair of brain injury (Matsumoto et al, 2024). The finding that PSA-NCAM levels are decreased after injury is unexpected, as usually injury is associated with increased expression of PSA-NCAM. They used Dcx-GFP mice as well as nestinCreERT2::NSE diptheria toxin mice in order to remove SVZ neuroblasts and show they are necessary for functional improvement. Analysis of this data was carried out with a well-thought through semi-automatic imaging and segmentation approach to quantify adhesion. They then used deep learning, mathematical modelling and quantification of directed versus entropic migration. Their use of common marmosets showed these mechanisms are also at play in primates.

Using these powerful approaches, the authors show that there are more non-adherent areas between newborn neurons than between neurons and astrocytes. They also find that even within adherent areas there are small “pores” of non-adherence. Then they show that brain injury increases the proportion of adherent areas and decreases pores between neuroblasts suggesting this may restrict repair. Endogenous neuraminidase can regulate adhesion by cleaving PSA-NCAM and therefore migration of SVZ neuroblasts. Decreasing Neu1 (neuraminidase) with knockdown massively increased emigration to injury and PSA-NCAM expression. Finally, neuraminidase inhibitors increased migration to injury.

Overall, this is a fascinating set of results using novel and powerful approaches which may have significant effects on the field. The role of reduced PSA-NCAM expression in chain migration in trauma is interesting and may help explain the relatively low level of repair and is thus interesting and important to consider. The findings point to a novel druggable target to increase endogenous neuroblast migration to injury—neuraminidase inhibitors.
